# Supplementary material for: Maternal death surveillance and response system reports from 32 low-middle income countries, 2011–2020: What can we learn from the reports?
Source: PLOS Glob Public Health. 2024 Mar 5;4(3):e0002153. doi: 10.1371/journal.pgph.0002153 (PMC10914274; doi:10.1371/journal.pgph.0002153)
Supplement: S1 Text — (DOCX) [file pgph.0002153.s001.docx]

# **Report content analysis items**

**National Reports on Maternal Death Surveillance**

**Case-study Data Collection Tool**

**Country ______________**

**Year of report issuance__________**

| **Reporting Period** (*when the* *deaths included in the reports occurred)* |
| --- |
|  |
| **Type of Report** *MDR/CEMD/MDSR/MPDR/MPDSR* |
|  |
| **Is this an annual or non-annual report?** *(If not annual, specify timeframe: biennial, triennial, quinquennial)* |
|  |
| **Coverage** *(national or subnational)* |
|  |
| **Population included in data collection** *(whole population, urban only, rural only)* |
|  |
| **Description of methods used to obtain information on maternal deaths** *(hospital records, verbal autopsy, vital records, health management information system)* |
|  |
| **Data completeness described by report authors** *(complete, mostly complete, incomplete, completion not mentioned; include examples of reasons for assessing the data as incomplete)* |
|  |
| **Was the standard ICD definition of maternal death included?** (The death of a woman of reproductive age (defined here as 12–49 years) from any cause related to or aggravated by pregnancy or its management either during pregnancy or within 42 days of pregnancy outcome, irrespective of the duration or site of the pregnancy)[28] *(yes, no)* |
|  |
| **Did the report include the number of perinatal deaths?** (infant deaths that occur at less than 7 days of age and fetal deaths with a stated or presumed period of gestation of 28 weeks or more)[28] *(yes, no)* |
|  |
| **Were both maternal death due to direct and indirect obstetric causes reported?** (Direct obstetric deaths result from obstetric complications during pregnancy, labor, or the puerperium from interventions, omissions, incorrect treatment, or from a chain of events resulting from any of the above. Indirect obstetric deaths are those that result from a previously existing disease or a disease that developed during pregnancy and was not directly related to direct obstetric causes, but was aggravated by the physiological effects of pregnancy)[28] *(yes, no)* |
|  |
| **Was the maternal mortality ratio (MMR) reported? If included, was MMR calculated correctly?** *(Refers to the MMR derived from number of deaths included in the report)* |
|  |
| **Was the denominator for MMR specified? How collected?** *(yes, specify; no)* |
|  |
| **Were deaths reported for the whole population?** *(yes, no, specify population excluded)* |
|  |
| **Were deaths reported by geographic areas (e.g., province, region, district)?** *(yes, no)* |
|  |
| **Were deaths occurring in health facilities reported?** *(yes, no)* |
|  |
| **Were deaths occurring in communities reported?** *(yes, no)* |
|  |
| **Was the legal framework described?** *(e.g., notifiable condition; confidentiality of reporting; protection of providers who report) (at least one legal provision described, specify)* |
|  |
| **Did the report include or mentioned the existence of a MDSR national policy?** *(yes, no)* |
|  |
| **Did the report mention milestones in MDSR implementation?** *(yes, specify; no)* |
|  |
| **Did the report mention the number of maternal deaths notified?** *(yes, no)* |
|  |
| **Did the report mention that a maternal death must be notified within a certain time period?** *(yes, specify; no)* |
|  |
| **Did the report describe the notification process, including use of zero reporting?** *(yes, no)* |
|  |
| **Did the report mention the number of maternal deaths reviewed?** *(yes, specify; no)* |
|  |
| **Did the report mention a notification rate?** *(yes, no)* |
|  |
| **Was the denominator data for notification rate included?** *(yes, specify; no)* |
|  |
| **Did the report include a description of the death reviews** – Who? How often? How done? *(specify)* |
|  |
| **Did the report mention the number of maternal deaths reviewed?** *(yes, no)* |
|  |
| **Did the report mention a review rate?** *(yes, no)* |
|  |
| **Was the denominator data for the review rate included?** *(yes, specify; no)* |
|  |
| **Were data analyzed by cause of death?** *(yes, specify; no)* |
|  |
| **Were data analyzed by age?** *(yes, specify age groups; no)* |
|  |
| **Were maternal death data analyzed by pregnancy outcome (i.e., live birth, stillbirth, abortion, other pregnancy loss, undelivered)?** *(yes, specify; no)* |
|  |
| **Were maternal deaths analyzed by non-medical contributing factors, such as the 3 Delays** *(yes, specify; no)* |
|  |
| **Were any other additional analyses done? (i.e., more than frequencies)** *(yes, specify; no)* |
|  |
| **Did the report include recommendations?** *(yes, no)* |
|  |
| **If recommendations were included, what was the quality of the recommendations (SMART criteria- specific, measurable, achievable, relevant, timebound)** *(yes, specify; no)* |
|  |
| **If recommendations were included, were recommendations addressing what levels? (community/facility/regional/national/other)** *(yes, specify; no, recommendations were general)* |
|  |
| **If recommendations were included, were they linked with issues identified by the reviews** *(yes, examples; no, not specifically linked to identified issues)* |
|  |
| **Example of recommendation(s) provided in report to address an identified issue(s)** *(narrative)* |
|  |
| **Did the report include any follow-up from any previous recommendations (showing country learning and improvements over time)?** *(yes, specify, no, first report, no)* |
|  |
| **Did the country have a national committee that coordinate the MDSR activities? If yes, does the report describe its composition?** *(yes, specify; no)* |
|  |
| **Did the country have sub-national committees that coordinate subnational MDSR activities? If yes, does the report describe its composition?** *(yes, specify; no)* |
|  |
| **Did the report mention the MDSR focal point/lead?** *(yes, specify; no)* |
|  |
| **Did the report include an executive summary or conclusions?** *(yes, specify; no)* |
|  |
| **Did the report include tables and figures?** *(yes, specify; no)* |
|  |
| **Did the report include maps?** *(yes, specify; no)* |
|  |
| **Did the report include surveillance tools, for example notification form, review form, verbal autopsy form?** *(yes, specify; no)* |
|  |
| **Other relevant appendices included** *(specify)* |
|  |
| **This report is a good example of…** *(narrative)* |
|  |

Acronyms: CEMD, confidential inquiries in maternal deaths; MDR, maternal death review, MDSR, maternal death surveillance and response; MMR, maternal mortality ration; MPDR, maternal and perinatal death review; MPDSR, maternal and perinatal death surveillance and response.

Reference:

World Health Organization. *International Statistical Classification of Diseases and Related Health Problems, Tenth Revision*. Geneva: World Health Organization; 1992.
