# Supplementary material for: Maternal death surveillance and response system reports from 32 low-middle income countries, 2011–2020: What can we learn from the reports?
Source: PLOS Glob Public Health. 2024 Mar 5;4(3):e0002153. doi: 10.1371/journal.pgph.0002153 (PMC10914274; doi:10.1371/journal.pgph.0002153)
Supplement: Attachment — Submitted filename: Response to Reviewers.docx [file pgph.0002153.s002.docx]

Rebuttal letter for PGPH-D-23-00536

Dear PLOS Global Health Editors,

On behalf of the co-authors and myself, thank you for the opportunity to revise and resubmit our manuscript entitled “Maternal death surveillance and response system reports from 32 low-middle income countries, 2011–2020: what can we learn from the reports?”. We would like to thank the Editor for considering our paper for publication. We are grateful for Editor’s and Reviewers’ time to give us very detailed comments and suggestions. We incorporated their feedback and responded to their questions in detail in the point-by-point responses. Below, we provide a response to each point raised by the editor and reviewers, with our responses in blue text.

**Editor Comments**
Journal Requirements:

1. Please review your reference list to ensure that it is complete and correct. If you have cited papers that have been retracted, please include the rationale for doing so in the manuscript text, or remove these references and replace them with relevant current references. Any changes to the reference list should be mentioned in the rebuttal letter that accompanies your revised manuscript. If you need to cite a retracted article, indicate the article’s retracted status in the References list and also include a citation and full reference for the retraction notice.

- We have reviewed the reference list and confirm that it is complete and correct.

2. We noticed that you used "unpublished" in the manuscript. We do not allow these references, as the PLOS data access policy requires that all data be either published with the manuscript or made available in a publicly accessible database. Please amend the supplementary material to include the referenced data or remove the references.

- We removed the reference to unpublished data on page 7 of the paper. This had referred to the Maternal Health Thematic Fund survey data, which is included in reference #5.

3. In the online submission form, you indicated that "Data are available from the CDC Division of Reproductive Health upon reasonable request". All PLOS journals now require all data underlying the findings described in their manuscript to be freely available to other researchers, either 1. In a public repository, 2. Within the manuscript itself, or 3. Uploaded as supplementary information. This policy applies to all data except where public deposition would breach compliance with the protocol approved by your research ethics board. If your data cannot be made publicly available for ethical or legal reasons (e.g., public availability would compromise patient privacy), please explain your reasons by return email and your exemption request will be escalated to the editor for approval. Your exemption request will be handled independently and will not hold up the peer review process, but will need to be resolved should your manuscript be accepted for publication. One of the Editorial team will then be in touch if there are any issues.

- Thank you for this clarification. As this was a qualitative thematic and content analysis, all data can be found in the country reports cited in this paper. No report includes individual or identifiable information. Some reports are available online, others were obtained through the UNFPA country offices and are internal documents produced by the country Ministries of Health. We could provide the library of reports in a separate repository, if we receive country concurrence from countries that only shared the reports with UNFPA country offices for the purpose of this analysis. Or we can deidentify all reports and include them in a repository. Alternatively, we could include all reports as references or list the reports as a supplemental material.

**Reviewer Comments to the Author**

**Reviewer #1:** Maternal death surveillance and response system reports from 32 low-middle income countries, 2011–2020: what can we learn from the reports?

General comment:
This a generally well researched and written paper that was needed. The status of implementation of MDSR system across countries was largely unknown except from research that have been done in few countries. This study provides a glimpse on how countries have accepted and made efforts to implement the system.

*Abstract*
The abstract is well written

*Background*
It is a well written

*Methods*
1. Can the authors say what type of study was this? Was it qualitative study or a mix quantitative-qualitative study?

- We added text on page 7 to note that this is a qualitative analysis.

2. Its not clear why the author decided to include the reports before 2011, while the MDSR system was initiated by the WHO in 2013

- Thank you for this comment. We decided to include reports prior to 2011 to cover a full 10-year period and to have the opportunity to compare reports published before and after the WHO’s MDSR guidance. To clarify, the MDSR system existed prior to 2013. WHO guidance was published to support scaling up the MDSR implementation in 2013.

3. It is reported that some countries were excluded since they did not meet the inclusion criteria, is it possible to mention the countries that were excluded? Do ethics allow this? I think it would be good for these countries to know why they were excluded so that they can improve on their reporting.

While it may seem of interest to name the countries whose reports were not included, we thought against such approach for a couple of reasons:

The main reason for exclusion was lack of description of reporting and review process, as stated in the methods. “Reports that investigated maternal health care without providing data on reporting or review of maternal deaths were excluded.“ Countries that do not include these details in their reports would easily self-identify and would benefit from learning how other countries include such descriptions.

All countries, including those whose reports were included in our analysis, would benefit from improvements in their reporting. The World Health Organization’s MPDSR Technical Working Group aims to develop a “standard” reporting template for national reporting and make it available on-line to all countries that would choose to use this reporting aid tool.

4. The author explained well what was assessed and the data extraction guide which was obtained from the WHO guidance on dissemination and reporting. It is not clear whether the WHO guidance provides guide for reporting each item. Are all the countries using this guide or does each country decide how to report each item?

The guidance does include a recommendation on core elements for reporting. We added a clarification in the text. The list is not intended to be comprehensive and the emphasis in the guidance is placed on providing feed-back to facilities/communities and on confidentiality of the information reported. More reporting elements were included in our synthesis based on the WHO MPDSR Technical Working Group feedback.

5. The authors should also explain how the recommendations were assessed. Was the SMART criteria or any other criteria used??

- We used the SMART criteria to assess recommendations and added text describing this on page 7.

*Results*
1. Line 215: Some countries reported to have “complete” data. How did these countries assess the completeness. It not clear in general how the countries assessed completeness of their data. Is there guide in the WHO guidance on how to do this?

- This is an excellent observation. The WHO guidance does not make a recommendation on how to assess completeness. We reported the self-described completeness included in the country reports. Countries describing their data as “complete,” used different methods of assessing data completeness that were not always fully explained in reports. We added clarification to page 10.

2. Figure 1 is not well visible

- Thank you for noticing. We have revised Figure 1.

3. The text in all other figures in blurred. The author can provide figures with better resolution

- From our end the figures do not appear blurred; this may be an issue in picture file after inclusion in the pdf builder. Per the journal instructions, we run the figures through the Preflight Analysis and Conversion Engine (PACE) digital diagnostic tool, and they all look very clear. We can also provide the figures in other than TIF format, if needed.

*Discussions*
1. It’s a well written discussion

2. It would benefit with more references to show what research on MDSR in different countries have reported

- We include references on MDSR systems and reporting as relevant to this study, including references for studies conducted in Senegal, Burkina Faso, Kenya, Malawi, and larger cross-country studies. While the MPDSR literature grew substantially in recent years, to our knowledge the reporting has not been studied. We added the following reference citing the scoping review conducted by Kinney and colleagues, which included 58 studies from 24 low- and middle-income countries that were published between 2004 and 2018:

“While the literature on MPDSR implementation is growing in LMICs, particularly in Africa, Kinney and colleagues [4] found that most studies focused on “tangible inputs from a service delivery lens,” did not systematically report on all steps of the surveillance cycle or on the system as a whole, and often overlooked the role of implementers and communication channels within MPDSR or between MPDSR and the health system as a whole. Strengthening the quality of national reports, intended to report on the entire surveillance cycle, its monitoring, and its iterative improvements, may be able to address some of the implementation gaps identified in the literature.”

3. The authors should also discuss on reporting on how the reports were created and stakeholders involved in making the reports. This is another crucial element in making sure the reports reflect the situation in the countries with no biases or political influence in the reports.

- Thank you for noting this important point. We added the following information on page 5 to address this comment: Reports are generated by in-country teams of health scientists and physicians, typically convened by the Ministry of Health to conduct a thorough review of the MDSR system and to provide recommendations for system improvement.

**Reviewer #2:** The authors reviewed MDSR reports from low-and-middle income countries (LMICs) to examine core content and identify how surveillance data and data dissemination could be improved to guide recommendations and track responses. A deductive content analysis of 56 MDSR reports from 32 LMICs was conducted. Among other findings, the authors found that slightly over half of the reports described their data as incomplete and reported deaths at both community and facility levels, while a third calculated review completion rates. Differences were observed in how notification and review rates were calculated.

This is a very important evidence contributing to strengthening MDSR.

The authors have described the methods used comprehensively and have presented the results appropriately.

It would be good to also show the observed differences by region for further exploration and future understanding in case of major differences in the observations.

- We understand that the reviewer is asking about the MDSR system differences by world regions. Since all but 7 countries sharing their reports with us were from sub-Saharan Africa, we did not think we could provide an accurate regional analysis. We added the following sentence under the study limitations: “Further, our analyses predominantly included reports from sub-Saharan Africa, which prevents us from examining regional differences.”
- Otherwise, I have no further comments.

Thank you for your consideration of our revised manuscript submission to PLOS Global Public Health. We look forward to hearing from you regarding our submission at your earliest convenience.

Sincerely,

Florina Serbanescu

Corresponding Author
