# Supplementary material for: Maternal death surveillance and response system reports from 32 low-middle income countries, 2011–2020: What can we learn from the reports?
Source: PLOS Glob Public Health. 2024 Mar 5;4(3):e0002153. doi: 10.1371/journal.pgph.0002153 (PMC10914274; doi:10.1371/journal.pgph.0002153)
Supplement: Attachment — Submitted filename: Response to reviewer 3.docx [file pgph.0002153.s004.docx]

| **Editor / Reviewer comments** | **Response** | **Changes made *(Whether a change was made, what the change was, and where it can be found in the clean version*** |
| --- | --- | --- |
| The manuscript obviously addresses an important public health issue in maternity care. My major problem is that the conclusion of the abstract is far too vague and is not giving the only conclusion which can be made of this review. My comment is repeated at the conclusion of main manuscript: the most important conclusion from this study must be the suboptimal functioning of the MDSR system, which therefore at present will have no or very limited impact of improving maternal health. In the background of the abstract, you wrote: Maternal Death Surveillance and Response (MDSR) systems generate information that aid efforts to end preventable maternal deaths". Unfortunately we have no clear evidence of this too bold statement, I therefore changed this in "may aid". It is not just all the paperwork written in this paper in a somewhat bureaucratic way, that will have any influence on maternal health. But I would agree that when the whole health system functions in all possible ways, MDSR may aid to improve maternal health. But this review by no way could convince anybody with experience in the field (I mean with his feet in the clay) because of the suboptimal functioning of MDSR could do anything, while and I agree in principle it could help, when all other parts of the system are in place and function well. This has to made clear in the manuscript  There are many more minor issues which can be found in the uploaded and edited manuscript. In my view it is better to give everywhere the numbers of the reports with % between brackets. One last issue: women give birth and pizzas are delivered and services exist in shops; in health facilities care is given | We want to thank the reviewer for the detailed comments and excellent observations. We feel that the manuscript has improved by incorporating the reviewer’s comments and addressing questions raised. We revised the abstract, discussion and conclusion.  We clarified that we are not assessing if the MDSR system has an impact on improving maternal health, but what is the content of MDSR reports and their potential role in improving functionality of the MDSR systems. The “bold statement” was made in the WHO guidance and other MDSR literature. We revised the language and added that MDSR “may aid” to improvements. We also added that learning from reports may allow to assess if the systems are suboptimal. We agree with the reviewer that suboptimal systems will not influence maternal health.  We removed or condensed some content from the results.  We addressed the other minor issues and added numbers next to percentages throughout the manuscript. Replaced “delivery” with “birth. | Per below |
| generating information by MDSR will not necessarily aid efforts to end preventable maternal deaths: it "may aid" | Agreed, added to the text. | Added “may aid” to the Abstract Background, line 59 |
| I would prefer the numbers with % between brackets everywhere in the manuscript | We edited the manuscript to include the number and corresponding percentage | All instances where the numbers did not precede the %; percentages included with brackets |
| stating that the data were incomplete, in my view, does not mean that not all deaths were captured, that would be incomplete identification or notification, in every MDSR or confidential enquiry there will always data that are missing.data | These statements were part of the reports that we reviewed. Some reports included sections on data quality assessments that described incomplete coverage (not all regions reporting), incomplete notification particularly of community deaths, incomplete details available for reviews, lack of details in summaries prepared after review, and absence of action plans. Few reports included a comparison of the number of deaths reported by MDSR with estimated numbers from external sources. We think it is important to show that countries are trying to assess completeness and data quality, which would strengthen the surveillance system if addressed. | No change |
| this, however, is the most important part of MDSR: act upon the recommendation and only that "may aid to reduce the numbers of maternal deaths” | We agree that one function of the annual reports is to provide accountability of the actions taken (response) | Added to the conclusion |
| This is a vague conclusion, while it can be easy concluded that there are many lessons to be learned from the substandard reports in the majority. This indicates less effects of MDSRs in general | We intuitively agree with the reviewer, however it was not within our scope to evaluate the MDSR systems of these countries. | We added in the Abstract Conclusion “Comprehensive reports can improve accountability and effectiveness of the system by providing feedback to MDSR stakeholders and information for action.” |
| services are to be found in shops or restaurants; in health facilities we hope to get health care or maternity care | Service delivery is often use when referring to maternal and child health care  <https://www.who.int/teams/maternal-newborn-child-adolescent-health-and-ageing/maternal-health/service-delivery-with-quality> | “services” replaced with “care” page 4, line 101 |
| there is no proof whatsoever that this is "paramount for reducing"; yes it may help if all factors of the cycle and the health system are in place |  | Removed “paramount” and replaced with “may help” page 4, line 101 |
| You really take the inner bend here: there is much more in between guidance, using data and improve maternal health: there is a huge gap between MSDR reports and what is happening in the labour rooms and during pregnancies in the community! | This sentence is about the MDSR technical working group and not about reports. We are stating that the WG “provides countries with support and guidance for using MDSR data” | Page 6, line 138 added “that may in turn help reduce” preventable maternal deaths |
| it is unclear how many reports were totally included: from here you would say 53+28=81 or 38+28=66 and from abstract 56! | We included 56 reports. Of those, 38 were provided by 24 MHTF countries and 18 (not 28, apologies for the typo) from non MHTF countries. There are 27 MHTF countries that answered they published periodic MDSR reports on the MHTF questionnaire, but only 24 countries issued reports that met the inclusion criteria. | Revised on page 7 line 163 “The remaining 18 reports included in our analysis issued in 2011–2020 were identified by WHO and its MDSR working group from LMICs that do not participate in the MHTF surveys.” |
| what other consistency than just this | Removed “across the data extracted” | Page 8, line 176-177  Each report underwent independent review by two researchers to verify consistency ~~across the data extracted~~, and a third researcher weighed in to resolve disagreements. |
| one would like to know whether the recommendations in the previous report were implemented completely, partially or no implementation at all; has that been done in your sub-analysis? | We agree with the reviewer that more details on the extent to which the recommendations are followed up is important. Details about how completely the recommendations were implemented were not included in the 12 reports. | We added to the discussion the following: |
| I don't understand the wording  "high-level"overview, which I would consider a fully detailed overview and therefore of a high level! | We revised the sentence | Page 11, line 225 “Over half (54%, n=20) of the reports including a description of policies did not mention whether reporting was mandatory…” |
| is the number of reports describing MDSR policies different from the numbers including a description? Very confusing | We revised the sentence | Page 11, line 228 …” and 5% (n=2) ~~of reports describing MDSR policy~~ specified mandatory and zero reporting.” |
| I find this very futile data and would doubt if differences here would have any effect on what we all aim: less deaths! Beware of making MDSR too bureaucratic, increasing gap between health offices and the groundfloor where you can make a difference | We revised the sentence | Revised result on page 11, lines 230-234: “Among reports that detailed MDSR policies, 41% (n=15) did not mention timeliness of notification. When reports did provide a timeframe in which maternal deaths must be notified, most mandated a 24-48 notification timeframe.” ~~the following intervals were noted: within 24 hours (21%, n=12), 24 hours for facility-based and 48 hours for community-based deaths (11%, n=6), 24-48 hours (2%, n=1), 24 hours for facility and 72 hours for community-based deaths (2%, n=1), 48 hours (4%, n=2), or 72 hours (2%, n=1).~~ |
| again all very trivial data | We removed the sentence | Page 11, lines 243-245. We removed the sentence “Other reports did not mention the committees at all (20%, n=11), a national committee was mentioned in 11 reports (20%), and a subnational committee was mentioned in seven (13%).” |
| Doesn't that make these reports without any value? | As the reviewer said, “there are many lessons to be learned from these reports.”  We present the gaps in these reports to inform on the need for better content | No change |
| which is important, but will not have direct effect on better maternal health | We are unable to say. It is encouraging that most reports included recommendations, but whether the recommendations improved maternal health in the country cannot be determined without well designed evaluations. Even in countries where maternal mortality has declined over time, the cause-effect relationship between MDSR recommendations and mortality decline needs to be assessed separately. | No change |
| women give birth, pizzas are delivered and women get care, services are in shops | Women give birth in Labor and Delivery Wards all over the world. Labor and delivery are different stages of the birth process, | Replaced “deliveries” with “births” |
| isn't the objective of the first ANC visit the conformation of pregnancy; I have practiced in many LIC for more than 40 years and never heard of a confirmation of pregnancy visit apart from the first ANC-visit! | We thank the reviewer for this observation. The theme captured from qualitative analyses was that women attend the first ANC visit to confirm pregnancy. Often, they only receive a confirmation of pregnancy and the opportunity for nutritional interventions, screening and diagnosis, and disease prevention is missed. | Revised sentence in Table 2, row 3: “A significant number of women present for confirmation of pregnancy early and measures should be put in place to link pregnancy confirmation visits with receipt of comprehensive ANC content (e.g., nutritional interventions, screening and diagnosis, and disease prevention).” |
| haven't we the ICD-MM classification? | Again, this is a quote, as it was stated in the country reports. And ICD-10MM is using the same codes as ICD-10. | No change |
| doesn't the same be true in government hospitals? | While the data quality is a challenge in all hospitals, this theme concerns coverage; private health facilities are not required to report to the health management and information system or surveillance systems in some countries. | No change |
| are regions generally not consisting of a number of districts? this is a strange order | That is correct, but it is rare to have a central blood bank in each district in LMIC. Some small regions may need to get blood from a neighboring region. And this is a quote from one report, the theme highlighted in other reports is low availability of blood. | No change |
| not only PPH! | Agreed, but this is a quote from one report | No change |
| data are plural | Agreed, but this is a quote. We corrected the grammar of the quote | Page 14, Table 2 cont. row 6: “At national level there should be an annual symposium at which the latest national data on maternal death [are] presented with appropriate analysis. |
| what is the difference with what you wrote in line 230: Most reports (66%, n=37) described their national MDSR policies, apart from 54%, n+30 | National policies provide the backdrop for the MDSR systems. A detailed system (identification and notification, reporting, review, analysis and response) was included in 30 reports. Our intention was to underline that many countries lack national coverage despite national system descriptions. | Rewording on Page 15, lines 291-293: “Over half of reports (54%, n=30) ~~described the country’s national~~ presented maternal death data from the MDSR system for the entire country, but maternal death data were only presented for a subset of reporting regions in 44% (n=26) of reports. |
| one then wants to know why? | Despite having national policies and a national system in place, some countries still struggle to enforce policies and implement the system in all regions. Lack of funds, human resources, adequate supervision and delays in reporting are reasons given for not being able to have deaths reported from all regions at the time an annual report is prepared. | Added to the Discussion |
| weren't e.g. notification criteria not the same in the national MDSR guide? | The WHO MDSR guide, which serves as the basis of national MDSR guides, did not define a notification rate. When countries used a notification rate, its definition and calculation varied. | no change |
| Edits made in Box 1 | We revised content of Box 1 | Antenatal care: Any ANC, number of visits.  Condition on admission to the hospital (e.g., in labor or not; with signs and symptoms of obstetric complications or not) some reports include “dead on arrival” or “unconscious at arrival.”  Direct and indirect complications during pregnancy and childbirth (obstetric hemorrhage, pregnancy induced hypertension, obstructed labor, uterine rupture, abortion, puerperal sepsis, other direct complications, indirect complications) |
| I cannot follow this sentence. Yes, sometimes you may think that the MDSR system is in an early phase and will become better in the future, but I miss here the main findings of the suboptimal functioning of the MDSR-system, whichtherefore will have an almost negligible effect on mipproving maternal health. | We added to the sentence | Page 19, lines 379-91: “Reduced coverage of maternal death notification and review could be interpreted as a reflection of the phase of MDSR scale-up rather than poor system performance but could also indicate poor implementation of scaled-up systems. Underperforming systems are likely to have an almost negligible effect on improving maternal health. |
| I haven't read this in the results | This is included at the bottom of page 15:  For reports that included a notification rate (29%, n=16), meaning the proportion of maternal deaths notified out of the total known or estimated from other sources, the denominator for this calculation came from a range of sources (Figure 4)….. | No change |
| and in this study | Added | Page 21, lines 434-436: Strengthening the quality of national reports, intended to report on the entire surveillance cycle, its monitoring and its iterative improvements, may be able to address some of the implementation gaps identified by our study and in the literature. |
| but also sub-Saharan Africa also has different regions | That is correct. However, the same themes emerge from reports across sub-Saharan Africa. And is a common grouping utilized by UN and World Bank organizations | No change |
| isn't that very unlikely giving the number of reports you could include? | The absence of a core section that reports on follow-ups from the previous report makes difficult to interpret if recommendations were implemented, to what extent, and resulting outcomes. We list this as a limitation of our study, but it is more a limitation of the content of the reports. | Page 23, line 456 Added to clarify:  “Without adding a core section that describes actions taken following the previous report makes difficult to interpret if recommendations were implemented, to what extent, and their resulting outcomes.” |
| the most important cocclusion from this study must be the suboptimal functioning of the MDSR system, which therefore at present will have no impact of improving maternal health. | We agree that functionality of MDSR systems in LMIC must improve. Some countries, however, credit their progress in reducing MM to their MDSR systems, even in the absence of a system evaluation. A recent Cochran review assigns 35% mortality reduction to well designed and implemented maternal death reviews, though it is based on a handful of studies meeting inclusion criteria (added reference 26). Our best conclusion is that MDSR could improve maternal health when it is accurately designed, implemented and evaluated for effectiveness. | Added:  Comprehensive reporting on MDSR may help the system in accomplishing its core functions of improving accountability and quality of care. Reports need to include detailed descriptions of the system’s key processes, particularly the response, the recommendations that have been addressed, to what extent, and if they are having the expected effect on maternal health. Ignoring suboptimal functioning of MDSR in national reports prevents countries in utilizing the system effectively and efficiently for reducing maternal deaths. |
